# The impact of telomere length on prostate cancer aggressiveness, genomic instability and health disparities

**DOI:** 10.1038/s41598-024-57566-1

**Published:** 2024-04-02

**Authors:** Ruotian Huang, M. S. Riana Bornman, Phillip D. Stricker, Ilma Simoni Brum, Shingai B. A. Mutambirwa, Weerachai Jaratlerdsiri, Vanessa M. Hayes

**Affiliations:** 1 Ancestry and Health Genomics Laboratory, Charles Perkins Centre, School of Medical Sciences, Faculty of Medicine and Health, University of Sydney, Camperdown, NSW 2006 Australia; 2 School of Health Systems and Public Health, Faculty of Health Sciences, University of Pretoria, Pretoria, 0084 South Africa; 3 Department of Urology, St Vincent’s Hospital, Darlinghurst, NSW 2010 Australia; 4 Endocrine and Tumor Molecular Biology Laboratory, Instituto de Ciências Básicas da Saúde, Universidade Federal do Rio Grande do Sul, Porto Alegre, Brazil; 5 Department of Urology, Sefako Makgatho Health Science University, Dr George Mukhari Academic Hospital, Medunsa, 0208 South Africa; 6 Manchester Cancer Research Centre, University of Manchester, Manchester, M20 4GJ UK; 7 Faculty of Health Sciences, University of Limpopo, Turfloop Campus, Sovenga, 0727 Limpopo South Africa

**Keywords:** Prostate cancer, Telomere length, Health disparity, Aggressive disease, African ancestry, Genomic instability, Cancer genomics, Urological cancer, Cancer genomics, Urological cancer

## Abstract

The telomere repetitive TTAGGG motif at the ends of chromosomes, serves to preserve genomic integrity and chromosomal stability. In turn, genomic instability is a hallmark of cancer—implicating telomere disturbance. Prostate cancer (PCa) shows significant ancestral disparities, with men of African ancestry at the greatest risk for aggressive disease and associated genomic instability. Yet, no study has explored the role of telomere length (TL) with respect to ancestrally driven PCa health disparities. Patient- and technically-matched tumour-blood whole genome sequencing data for 179 ancestrally defined treatment naïve PCa patients (117 African, 62 European), we assessed for TL (blood and tumour) associations. We found shortened tumour TL to be associated with aggressive PCa presentation and elevated genomic instabilities, including percentage of genome alteration and copy number gains, in men of African ancestry. For European patients, tumour TL showed significant associations with PCa driver genes *PTEN*, *TP53*, *MSH2*, *SETBP1* and *DDX11L1*, while shorter blood TL (< 3200 base pairs) and tumour TL (< 2861 base pairs) were correlated with higher risk for biochemical recurrence. Concurring with previous studies linking TL to PCa diagnosis and/or prognosis, for the first time we correlated TL differences with patient ancestry with important implications for future treatments targeting telomere dysfunction.

## Introduction

Prostate cancer (PCa) is the second most prevalent cancer in men, accounting for 1.41 million cases and 380,000 deaths worldwide in 2020^1^. Notably, PCa incidence and mortality rates vary dramatically across the globe, leading to significant ancestral health disparity. Specifically, Asian countries exhibit the lowest incidence and mortality rates, European ancestral countries present overall with the highest incidence rates, while mortality rates are highest for African ancestral Caribbean and Sub-Saharan Africa, almost threefold greater than global averages^2^. Although data is fragmented, PCa incidence rates are reportedly on the increase in Sub-Saharan Africa^3^, where PCa was reported as the leading cause of male-related cancer deaths in 2020^4^. Southern Africa is no different, it has around two times greater risk of presenting with aggressive disease than African Americans^5^. Compared to their European counterparts, African American men are more likely to be diagnosed at an earlier age with higher prostate-specific antigen (PSA) levels and are more likely to experience aggressive disease, including post-surgical relapse^6^. Taken together, it is well-established that African ancestry is a significant risk factor for PCa and aggressive disease, which is attributed to a combination of environmental, socioeconomic and genomic (biological) factors^7,8^.

Understanding the role of genomics in PCa health disparities has, however, been limited by access to African-relevant data, which is further perpetuated by a lack of technically and computationally matched ancestral data. To overcome these limitations, in 2022 we generated deep sequenced PCa whole genome data for 179 men of African and European ancestry presenting with largely advanced treatment naïve disease^9^. Analysed using a single technical and analytical pipeline, we observed significant genomic disparities. Compared to European data and although presenting with a larger number of germline variants^9^, African men were less likely to present with a known PCa risk allele^10^ and/or pathogenic germline variant^11^. Additionally, African-derived tumours presented with a larger spectrum and longer-tail of cancer drivers, including notable differences in commonly altered PCa genes such as a higher frequency of *SPOP* mutations and *PTEN* deletions, and lowered frequency of *TMPRSS2-ERG* fusions. Significant genome-wide tumour differences included an elevated tumour mutational burden (TMB), percentage genome alteration (PGA) and number of mutational signatures^9^. Furthermore, through integrative clustering for all types of somatic variants (single nucleotide variants (SNVs), small insertions/deletions (indels), copy number alterations (CNAs), and structural variants (SVs)) we described a novel PCa molecular taxonomy, named Global Mutational Subtype (GMS), where GMS-B and -D appear exclusive to African-derived tumours. What was lacking from these analyses was the determination of telomere length (TL) and its role with regards to PCa health disparities, disease presentation and genomic instability.

A human telomere is a short GC-rich DNA sequence on the 3’ and 5’ ends of a chromosome and consists of hexamer motif (TTAGGG)^12^, bound and protected by the Shelterin protein complex^13^. A human genome generally has a telomere length of 5–15 kilobase pairs (kbp) which shortens with age at 27 bp per year^14,15^. During the cell division, telomere shortening preserves genomic integrity and stability from chromosomal loss, through incomplete cell replication at chromosome ends by DNA polymerase^16^. Tumour cells have the capacity of unlimited proliferation, which is mainly achieved by telomere lengthening to avoid apoptosis^17,18^. Tumour telomeres are balanced by shortening and lengthening, which were found to be overall shortened distinguishably in European PCa patients than in normal controls^19^. Tumour TL and TL ratios (tumour TL/blood TL) of European PCa patients were observed to be negatively associated with PSA level and genomic instabilities, including PGA, SV and SNV, despite no focus on the ethnic disparity of TL data^19^. Considering the lack of African-relevant data, here we investigated for possible correlations of TL with PCa features within our previously sequenced whole genome data for 179 men representing either African or European ancestries and presenting with largely treatment naïve aggressive disease. Ultimately, we provide the first evidence for the contribution of TL and associated genomic instabilities and clinical presentations in PCa health disparities.

## Methods

### Whole prostate cancer genome dataset and patient clinicopathology

Our study included 179 PCa patients derived from published whole genome sequenced (WGS) blood-tumour matched data aligned to the human reference genome hg38 with alternative contigs and germline and somatic variant calling and annotation derived from a single technical and analytical workflow^9^. In brief, mean blood and tumour germline and tumour genome coverage achieved was 46X (range 30 to 98), 90X (range 28 to 139) and 90X times (range 28 to 139), respectively, while tumour purity ranged from 13 to 88% (mean 48%). Tumour genome features and instabilities were defined as clonality, genome alteration proportion (or PGA), the proportion of total somatic SNVs and indels per megabase of DNA (or TMB), somatic aberrations including SNVs, indels, SVs, regional gains and losses, as well as global PCa taxonomy (or GMS), tumour mutational signatures defined as single base substitutions (SBS), double base substitutions (DBS), indels (ID) or SVs, and PCa-related driver genes (n = 35).

Patients included 121 South Africans recruited at biopsy (all treatment-naïve), and 53 Australians and 5 Brazilians (recruited at surgery, with a single patient having received prior treatment). Patient ancestral substructures were derived from over 7 million germline SNVs using fastSTRUCTURE v1.0 tool^20^, with 117 defined as ‘African’ (98% African ancestral fractions, 116 South Africans and 1 Brazilian) and 62 as ‘European’ (53 Australians, 5 South Africans and 4 Brazilians) allowing up to 3% African ancestral and 26% Asian contributions. While clinicopathological presentation was similar between the ancestries, with 63.2% and 85.5% of African and European patients presented with International Society of Urological Pathologist (ISUP) group grading $\ge$ 3, respectively, African patients presented on average 5-years later, with significantly elevated PSA levels ($\ge$ 20 ng/ml), concurring with previous reports^5^. Due to availability of extensive clinical follow-up (range 37.4 to 214.3 months) for all the Australian patients, further outcomes data including biochemical relapse and/or a PCa-related death have been recorded and made available. Cohort clinicopathological features are summarised in Supplementary Table S1.

### Telomere length prediction

To generate the most reliable TLs for further investigations, the stability and validity of two commonly utilised, yet alternative TL estimation tools, namely TelSeq and Computel were compared. The pipelines of TelSeq and Computel are shown in Supplementary Figure S1. TelSeq, the first and most widely used computational TL measurement, computed tumour and blood alignment data for TL in kbp (1000 bp) and analysed telomeric read counts (n = 0–16), and telomeric reads with GC composition between (40 + n*2%)−(42% + (n + 1)*2%) (n = 0–9). The threshold of the abundance of telomeric repeats was set as default at k = 7. In brief, Telseq estimated real telomere sequences as the reads with at least seven telomeric repeats (k ≥ 7) with GC composition between 48 and 52% (n = 4)^21^. Conversely, Computel generated accurate TL results with no compulsory requirement of the alignment of a human reference genome, which calculated TL using a relative coverage of reads, deriving from a specific telomeric coverage of sequences mapped to the telomeric reference and the unmapped sequences coverage^22,23^. For Computel, we converted each alignment data into forward and reverse FASTQ reads. The FASTQ data were generated using a 20-nucleotide read length, six telomeric patterns for each base chosen as the starting nucleotide, and the minimum 10-bp seed length as default.

Using our WGS data resource, we observed a strong correlation for both blood TL (BTL, *P* = 0.833) and tumour TL (TTL, *P* = 0.922) between the two tools (Supplementary Figure S2A). Notably, both BTL and TTL estimates were significantly longer using TelSeq over Computel (*P* = 2.864e−12 to 2.204e−04). This is as expected, as Telseq allows interstitial telomeric reads (ITRs), which are not telomeres containing the telomeric motif localising at intrachromosomal sites, with 48–52% of GC composition to pass through the filter where Computel gates ITRs more strictly based on the inherent algorithms^23^ (Supplementary Figure S2B). BTL and TTL results from TelSeq were chosen for the subsequent analyses, while Computel was used for further analytical validations.

### European validation cohort

Publicly available whole genome derived tumour-blood paired TL data, with matched clinically relevant data, was sourced for 341 PCa treatment naive assumed European ancestral patients derived from five studies, three out of the United States, one from Canada and one from Germany^19^. Data source repositories include phs000447.v1.p1^24^, phs000330.v1.p1^25^, EGAS00001000400^26^, phs000178.v11.p8^27^, EGAS00001000400^28^. All TLs had been estimated using TelSeq (v0.0.1) from BAM files.

### Statistical analysis

To test clinical correlations of blood and tumour TLs with genomic and clinical PCa features by ethnicity, we performed a series of correlation tests, hypothesis tests and visual plots. Spearman tests examine a significant correlation between two non-normalised numeric variables with *P*-value < 0.05 regarded as statistically significant. For European patients, including the validation cohort, Kaplan–Meier survival curves were drawn for relapse-free and metastasis-free probabilities with optimal cut-off of shorter relapse and metastasis groups, followed by a log-rank test for significance at 0.05. Note, due to lack of follow-up time of metastasis for the validation cohort, only survival curve for relapse-free probability is shown, while follow-up clinical data was unavailable for our African patients. Group specific BTLs and TTLs medians with standard deviations and ranges are summarised in Supplementary Table S2. Mann*–*Whitney U test was used for non-parametric tests. Linear regression analysed data with two or multiple variables to show their associations. One-way ANOVA analysed the difference of means and possible correlations within multiple groups, which was used for age adjustment among variables in following analyses. Linear regression and one-way ANOVA analyses were all performed with age adjustment. Multiple hypothesis correction of *P*-values using Benjamini–Hochberg correction, presented as false discovery rate (FDR). All significant data were plotted on RStudio v2022.12.0 + 353.

### Ethics approvals and consent to participate

All deidentified data used in this study originated from published works derived from the SAPCS and St Vincent’s Hospital Garvan Institute Bioresource^9^, where all individuals provided informed consent to participate. In brief, patient recruitment for the SAPCS was performed under approval granted by the University of Pretoria Faculty of Health Sciences Research Ethics Committee in South Africa (with US Federal wide assurance FWA00002567 and IRB00002235 IORG0001762; HREC#43/2010), for the St Vincent’s Hospital Garvan Institute Bioresource in Australia by St Vincent’s Hospital HREC (SVH/12/231) and in Brazil by the Grupo de Pesquisa e Pós-Graduação (GPPG) Scientific Committee and Research Ethical Commission (20160539). Data generation and analyses were performed under appropriate fully executed Material Transfer Agreements (MTAs) and/or Data Sharing Agreements (DSAs), between the University of Pretoria, Garvan Institute of Medical Research or Universidade Federal do Rio Grande do Sul and the University of Sydney, with further ethics approval for genomic interrogation granted by the St. Vincent’s Sydney HREC (#SVH/15/227). This research conformed to the principles of the Helsinki Declaration.

## Results

### Telomere lengths and age by ancestry

**Figure 1:**
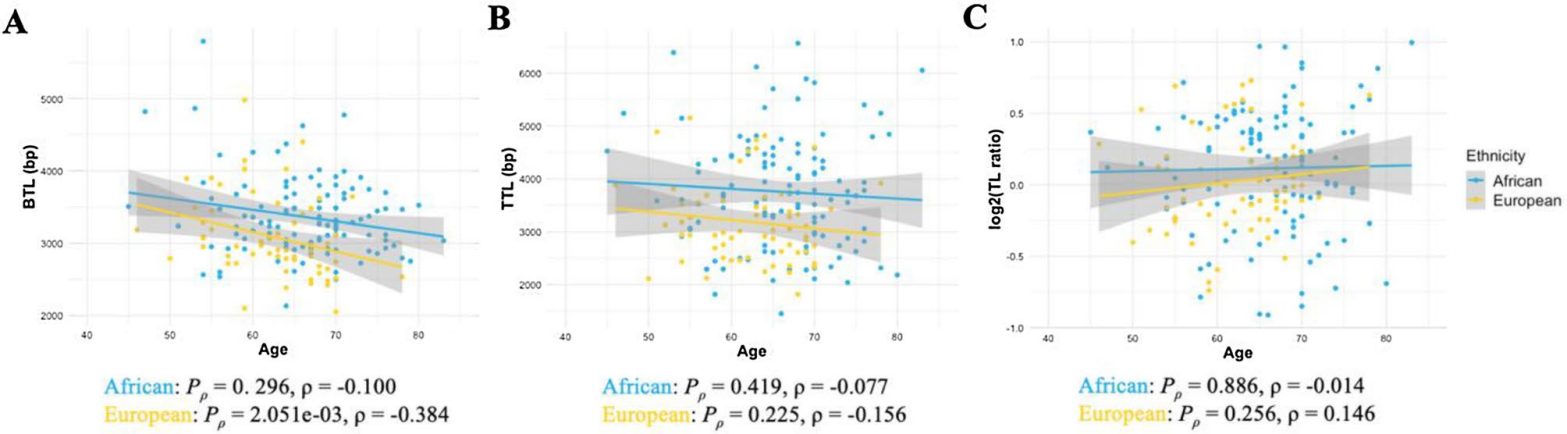
Correlations of BTL (**A**), TTL (**B**), and TL ratios (**C**) with age (40–85) for African (n = 112) and European (n = 62) PCa cases. *P*-values from Spearman’s correlation.

As expected, BTL and TTL estimates were shortened with older age in both ancestral groups (correlation coefficient ρ = − 0.384 to − 0.077) (Fig. 1A,B). BTLs showed significantly negative correlations with age in Europeans (*P* = 2.051e−03, ρ = − 0.384), although insignificant, European TL ratio indicated a positive correlation with age (*P* = 0.256, ρ = 0.146), while no significance was observed for African patients (*P* = 0.296 to 0.886) (Fig. 1A–C). Our European data concurs with ancestrally-matched validation data for all TL measurements and direction of correlation (Supplementary Figure S3), including a significant negative correlation between BTL and age (*P* = 8.172e−08, ρ = − 0.294). Age has been a defined confounder of TL shortening, and thus, the subsequent ANOVA and linear regression analyses were performed after adjusting for age.

### Telomere lengths and sequencing artifacts

The one-way ANOVA of TL determined the impact of genomic biases and errors when considering sequencing coverage (BTL and TTL), tumour purity (TTL only) and ploidy (TTL only) (Supplementary Table S3). We found that BTL and TTL were not correlated with sequencing coverage, and TTL showed an insignificant correlation with tumour purity and ploidy. Investigating all the variables in the analysis (sequencing coverage, tumour purity and ploidy), TL still showed an insignificant correlation of TelSeq results of TTL with all sequencing impacts, with similar *P*-values, suggesting that sequencing artifacts were unlikely to drive TL differences observed.

### Telomere lengths by site and ancestry

**Figure 2:**
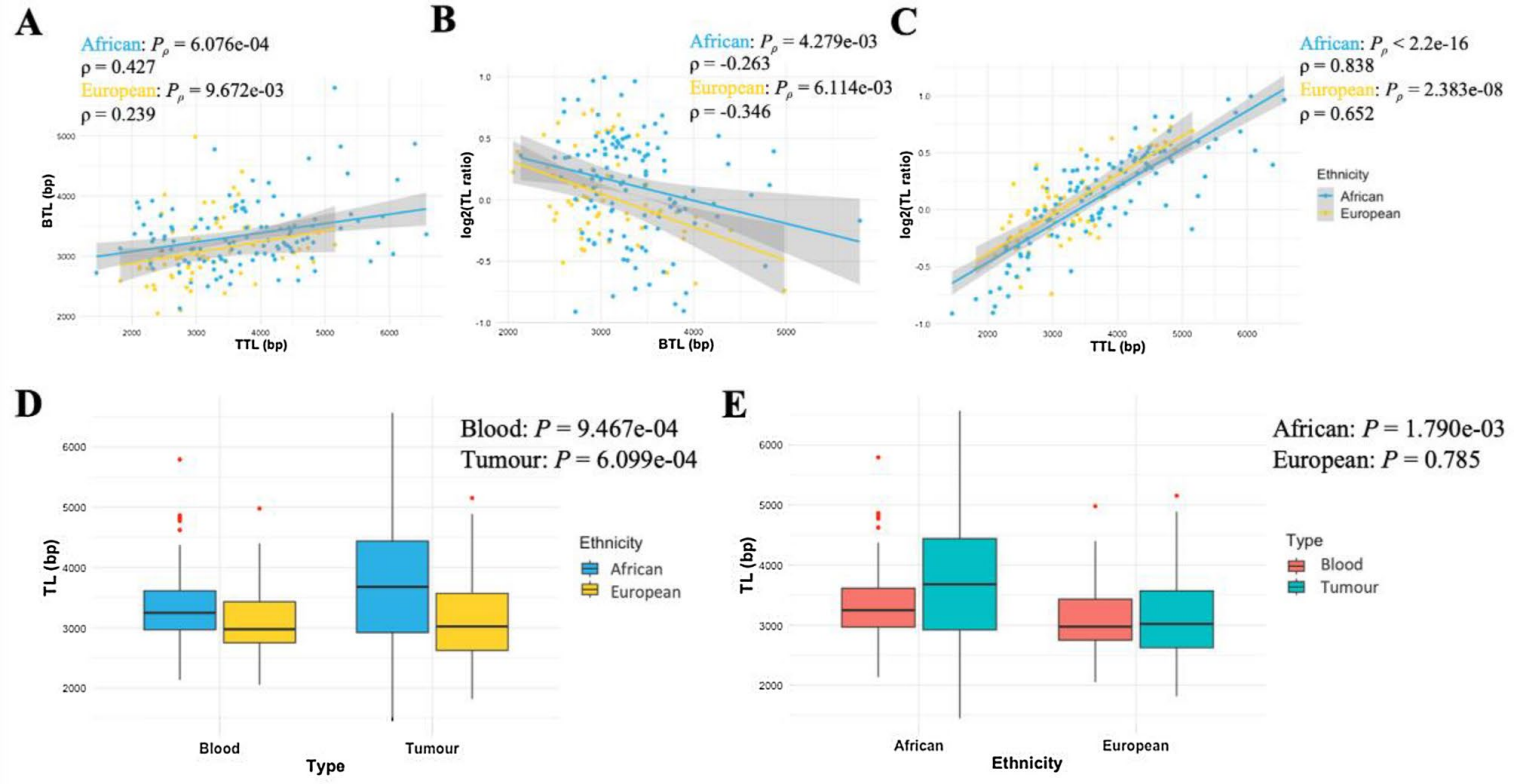
Spearman’s correlations of BTL and TTL estimates (**A**), TL ratios in log_2_ transformation and BTL (**B**), and TL ratios in log_2_ transformation and TTL by ancestry (**C**). Comparisons of African and European TL in blood and tumour samples (**D**) and those of BTL and TTL in African and European cohorts (**E**). *P*-values from Mann–Whitney U Test.

BTL and TTL estimates of African and European men were significantly correlated (*P* = 6.076e−04 and *P* = 9.672e−03, respectively; Fig. 2A), while TL ratios were negatively correlated with BTL (ρ = − 0.263 to − 0.346; Fig. 2B) and positively correlated with TTL (ρ = 0.652 to 0.838; Fig. 2C). The direction, slope and significance of these correlations were further validated in our public European cohort (Supplementary Figure S4). African men had significantly longer BTLs and TTLs than European men (*P* = 9.467e−04 and *P* = 6.099e−04, respectively; Fig. 2D). While there was no significant difference between BTLs and TTLs in Europeans, TTLs were profoundly longer in Africans (*P* = 1.790e−03; Fig. 2E). Of note, 34 out of 62 European PCa patients (54.8%) had shorter TTL than BTL, whereas shorter TTL was observed in 44 out of 117 African men (37.6%). This might suggest a higher duplication rate observed in African-derived tumours^9^.

### Telomere lengths and clinical presentation by ancestry

**Figure 3:**
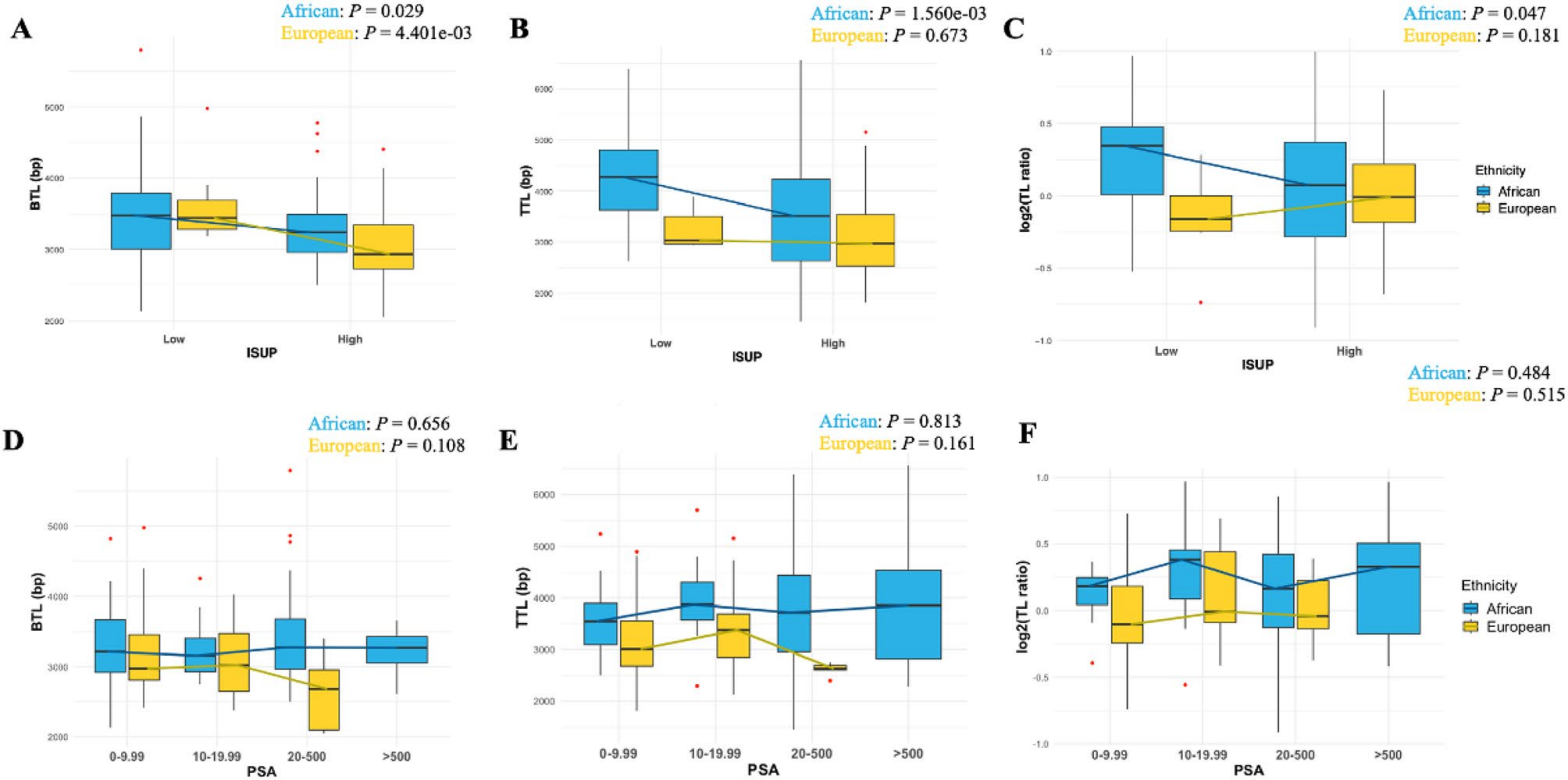
Correlations of BTL (**A**), TTL (**B**) and TL ratios (**C**) between low (1–2) and high (3–5) ISUP Grading Groups in African (n = 115) and European (n = 62) cohorts. *P-*values are from one-way ANOVA with age adjustment. Correlations of BTL (**D**), TTL (**E**) and TL ratios (**F**) with the following PSA levels (ng/mL): 0–9.99, 10–19.99, 20–500, and > 500 in Africans (n = 114) and Europeans (n = 62). *P*-values from one-way ANOVA with age adjustment.

BTLs revealed significant differences between patients presenting at diagnosis (South African) or surgery (Australian and Brazilian) with low and high ISUP grading group for both ancestries (African *P* = 0.029 and European *P* = 4.401e−03; Fig. 3A). However, further analysis of the European validation cohort showed no association (Supplementary Figure S5A). While only TTL estimates and TL ratios among Africans indicated significant shortening between ISUP grading groups, with higher ISUP $\ge$ 3 associated with shorter TTLs and decreased TL ratios (*P* = 1.560e−03 and *P* = 0.047, respectively; Fig. 3B,C). Conversely, no difference was found for tumours derived from European men, concurring with pubic data (Supplementary Figure S5B,C). Irrespective of ancestry, BTL, TTL and TL ratios were not correlated with PSA level at diagnosis (Fig. 3D–F), while in the European validation cohort (Supplementary Figure S5D–F), TTL is significantly correlated with PSA levels (*P* = 0.021).

### Telomere lengths and clinical outcomes in European cases

**Figure 4:**
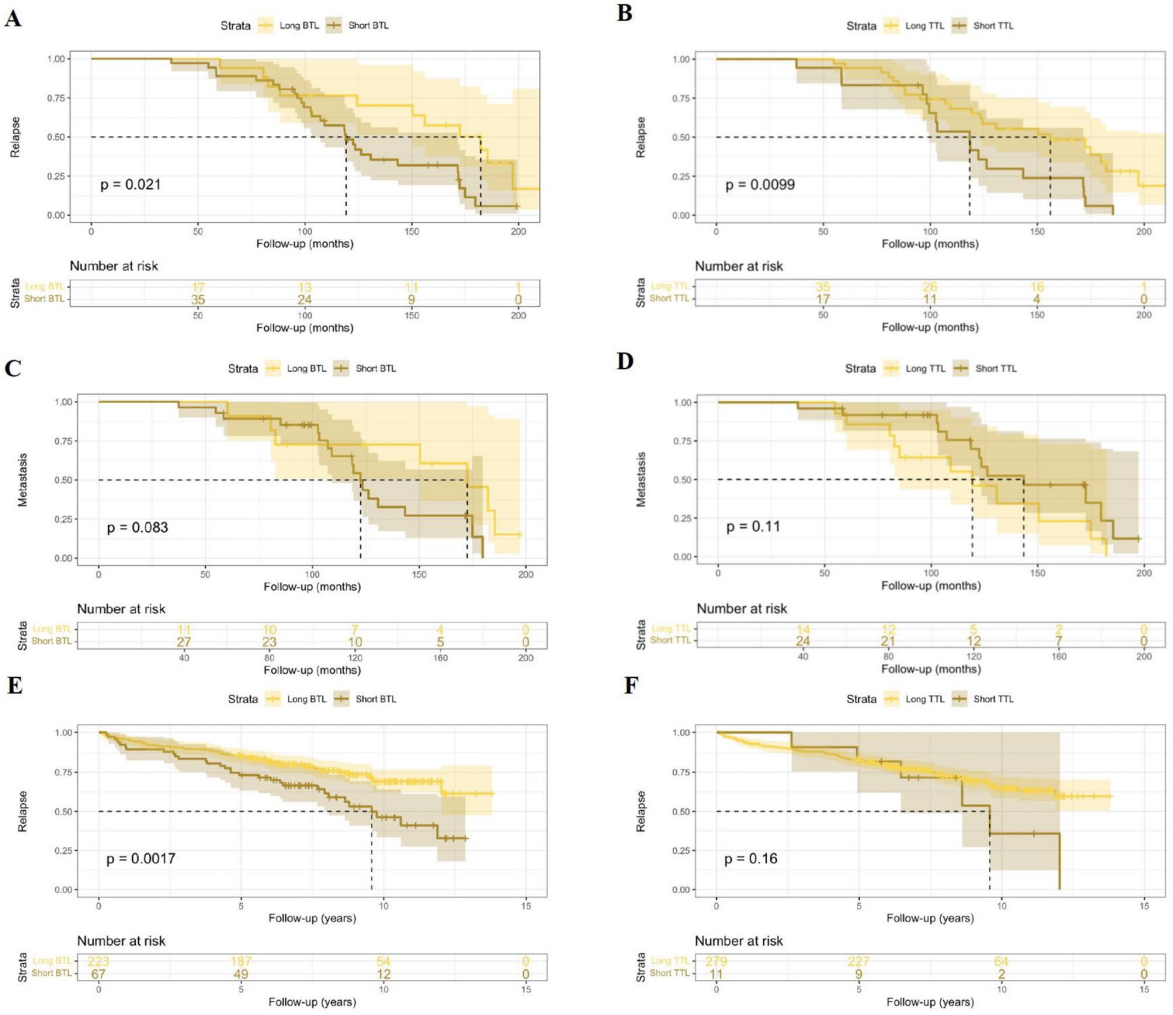
Kaplan–Meier survival curves for relapse-free probability over follow-up time (months) by BTL (**A**, 3126.568 ± 533.394 bp) and TTL (**B**, 3177.898 ± 773.048 bp) among 53 European PCa patients from our study (128.736 ± 44.355 months). Kaplan–Meier survival curves for metastasis-free probability over follow-up time (months) by BTL (**C**, 3099.618 ± 484.468 bp) and TTL (**D**, 3136.519 ± 823.521 bp) among 39 European PCa patients from our study (118.551 ± 42.191 months). Kaplan–Meier survival curves for relapse-free probability over follow-up time (7.347 ± 3.133 years) by BTL (**E**, cut-off = 3900 bp) and TTL (**F**, cut-off = 2000 bp) among 290 European PCa patients from the validation cohort.

We further sought to determine if TL was correlated with clinical outcomes, defined as biochemical recurrence (BCR) or metastasis, in men of European ancestries. Here we split the patients into short and long TLs groups defined as short/long TL by bisecting the TLs range until an optimal *P*-value was obtained^19^. The cut-off for TLs is tailored for each study to optimise the survival differentiation and is therefore not universal across different studies. European PCa patients with shorter BTLs (< 3200 bp) and TTLs (< 2861 bp), were at greater risk for earlier BCR (*P* = 0.021 and *P* = 0.0099, respectively; Fig. 4A,B), while no statistical association was found between BTL or TTL and metastasis (Fig. 4C,D). Having access to BCR data for 290 PCa patients from the European validation cohort, we concur that shorter BTLs (< 3900 bp) are correlated with earlier relapse (*P* = 0.0017; Fig. 4E), while not significant shorter TTLs (< 2000 bp) were more likely to be associated (*P* = 0.16; Fig. 4F).

### Tumour telomere lengths and associated genomic features

**Figure 5:**
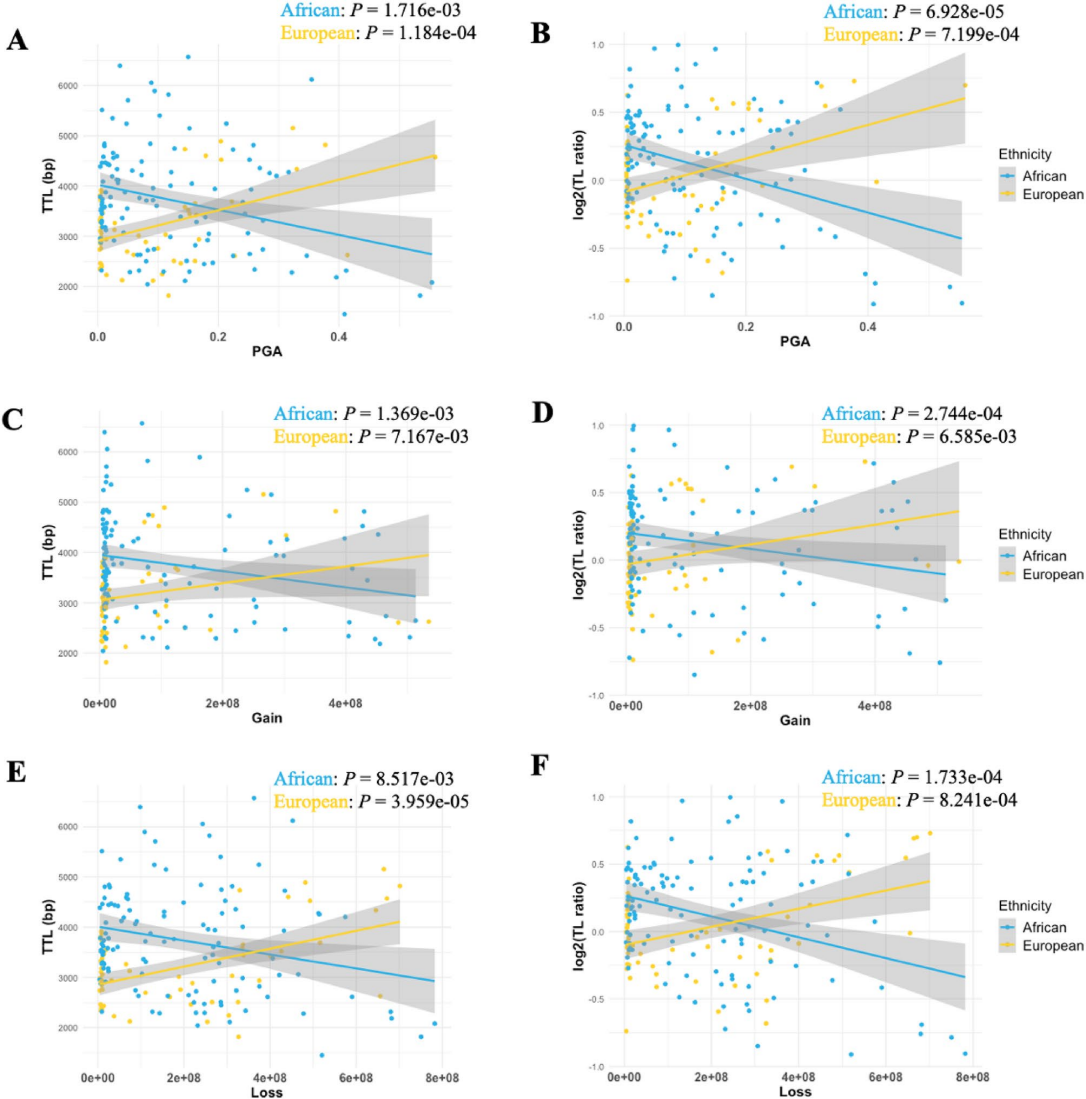
Linear regression of TTL and TL ratio in log2 transformation of African (n = 117) and European (n = 62) cohorts with genomic instabilities: PGA (**A**, **B**); Gain (**C**, **D**); Loss (**E**, **F**). *P*-values from one-way ANOVA with age adjustment.

Significant differences in TTL and TL ratios were observed for 117 Africans and 62 Europeans when correlated for 48 tumour genomic features, including the top 35 cancer driver genes. Strikingly and excluding for somatic SVs, clonality, SBS and ID, all TTLs and TL ratios were significantly associated with increased genomic instabilities, including PGA, TMB, somatic SNV, somatic indel, Gain, Loss, GMS, DBS and SV in African patients (all *P*-values ≤ 0.037, Supplementary Table S4). In the European validation cohort, we found TTL to be significantly correlated to PGA, somatic SNV and indels, with TL ratio additionally correlated with somatic GRs (all *P*-values < 0.05, Supplementary Table S5). After age and *P*-value adjustment, we found PGA, copy number gains and GMS to be correlated with TTLs and TL ratios, irrespective of patient ancestry, while SV was only associated with African TTLs and copy number losses were correlated with TL ratio of Africans and TTL and TL ratio of Europeans (Table 1), with significant , although the direction of the correlation was positive for European and negative for African ancestral tumours (Fig. 5). For our recently described PCa taxonomy (GMS), TTL and TL ratio significantly differed among all patients representing one of the four ancestrally relevant subtypes (GMS-A to -D). For impacted PCa driver genes, we found *SETBP1* tumours to be highly correlated with TTLs and TL ratio in both Africans and Europeans, while *MSH2* and *DDX11L1* tumours were associated with TTLs and TL ratios when derived only from European patients. Further comparisons unique to European derived tumours, included *PTEN* and TP53 associated with TTLs and *STK19* with TL ratios, while for African derived tumours only *FOXA1* was significantly correlated with TL ratio.

**Table 1:** Associations between 48 genomic features, including 35 driver genes, with age adjustment, and tumour TL and ratio using FDR by ancestry.

Genomic impacts	African (n = 117)		European (n = 62)	
	TTL	TL ratio	TTL	TL ratio
PGA	**0.021**	**0.032**	**1.137E−03**	**9.994E−04**
TMB	0.092	0.156	0.880	0.442
Somatic SNV	0.092	0.156	0.880	0.556
Somatic indel	0.105	0.156	0.810	0.092
Somatic SV	0.507	0.410	0.737	1.000
Gain	**0.021**	**0.032**	**0.038**	**0.012**
Loss	0.066	**0.036**	**4.752E−04**	**9.994E−04**
GMS	**5.232E−06**	**1.670E−08**	**1.565E−06**	**2.069E−08**
Clonality	0.642	0.478	0.658	0.429
SBS	0.356	0.502	1.000	1.000
DBS	0.066	0.156	0.724	1.000
ID	0.078	0.314	1.000	0.817
SV	**0.031**	0.052	0.773	0.986
*TMPRSS2*	0.336	0.448	0.773	0.998
*ERF*	0.356	0.410	NA	NA
*RB1*	0.797	0.790	0.175	0.283
*PTEN*	0.109	0.220	**0.018**	0.116
*FAT1*	0.587	0.502	0.810	0.715
*THADA*	0.263	0.328	0.724	0.970
*LSAMP*	0.975	0.571	1.000	1.000
*FOXA1*	0.109	**0.032**	0.724	0.429
*TP53*	0.077	0.156	**0.019**	0.092
*KMT2C*	0.066	0.156	0.756	1.000
*SPOP*	0.174	0.168	0.091	0.092
*MSH2*	1.000	0.922	**2.621E−03**	**2.138E−03**
*BRAF*	0.222	0.314	0.545	0.995
*MYC*	0.672	0.922	0.756	0.995
*ZMYM3*	0.222	0.456	0.658	0.998
*SETBP1*	**0.021**	**0.036**	**1.234E−06**	**2.520E−08**
*ZFHX3*	0.418	0.835	1.000	1.000
*CDK12*	0.410	0.410	0.756	0.429
*DDX11L1*	0.078	0.083	**2.720E−05**	**5.360E−04**
*STK19*	0.867	0.410	0.485	**0.048**
*NCOA2*	0.475	0.410	0.756	0.454
*PCAT1*	0.672	0.410	0.992	1.000
*MSH6*	NA	NA	1.000	1.000
*PAPSS2*	NA	NA	0.480	1.000
*MTCH2*	0.945	0.743	1.000	1.000
*ATR*	0.672	0.922	NA	NA
*BRCA1*	0.156	0.190	NA	NA
*POLE*	0.389	0.502	NA	NA
*TP53BP1*	0.867	0.922	NA	NA
*ATM*	0.066	0.156	NA	NA
*BRCA2*	0.092	0.156	NA	NA
*ERCC5*	0.456	0.593	NA	NA
*ERF*	0.356	0.410	NA	NA
*PIK3CA*	0.092	0.168	NA	NA
*APC*	0.389	0.156	NA	NA

False discovery rate (FDR) from *P-*values in one-way ANOVA. Driver genes included coding driver data, non-coding driver data, significantly recurrent breakpoint data, and gene-level copy data including recurrent deletion and amplification. *NA*: data not available.

Significant values are in bold.

## Discussion

Overall, we observe that men of African over European ancestry present with longer BTLs, which concurs with data for African Americans^29^. Notably, the rate of telomere shortening with age (also known as ‘weathering’) is less pronounced for our southern African *versus* European Australian patients. While the latter finding may contrast with the expectation that elevated exposure to socioeconomic stressors would accelerate biological aging and as such age-associated telomere shortening in our African cohort^30^, a 2019 study showed low socioeconomic status to be associated with a greater Black-White difference in age-related BTLs (5.66% longer in Black Americans), in contrast to individuals at higher socioeconomic status (2.33% longer in Black Americans)^29^. Although longer BTLs were initially marginally associated with increased PCa risk^31^, more recently shorter BTLs have been associated with aggressive PCa and worse prognosis^32^, with further confirmation for African American patients^33^. After adjusting for age, we found shorter BTLs to be significantly associated with aggressive disease presentation in both ancestral groups, although notably pronounced for men of European ancestry. However, we should caution that we were unable to validate the latter association using public data. While clinical follow-up data was not available or inconclusive for the African cohort, well-characterised follow-data was available for the European patient data. Here we found shorter BTLs (< 3200 bp) to have a strong correlation with worse prognosis after surgery and validated in our larger public-derived resource (< 3900 bp). Significantly correlated with aggressive disease presentation and disease relapse, our study implicates BTL as prognostic biomarker for long-term PCa surveillance.

In contrast to BTL, African American men presented with shorter TLs than their European counterparts when derived from benign or non-cancerous formalin-fixed prostate tissue assessed using a quantitative targeted approach^34^. While assessing TLs from fresh prostate tumour tissue, here we found tumours from southern African men to present with longer telomeres. Furthermore, we found shortened African derived TTL to be associated with higher ISUP grading group or more aggressive disease at diagnosis, indicating that TL shortening is involved or even promotes PCa carcinogenesis in African men. While not associated with worse histological presentation, European PCa patients with shortened TTL showed higher risks for earlier BCR onset, although not reaching significance in our validation cohort. We speculate if shortened TTL could have substantial potential as a target for aggressive PCa therapy in African men and a prognostic biomarker of relapse in European men. Possible limitations of our study include the systematic bias of algorithms and assumption of diploidy when using the TelSeq and Computel methodologies, resulting in an overestimation of TTL as a result of polyploidy^35^, with further potential impact created by varied tumour purity and sequencing coverages. Although no associations were found between TTL and all potential confounding variables tested, we appreciate that tumour heterogeneity, including our previous finding that African tumours presented with a longer-tail of cancer drivers^9^, as well as our relatively small sample sizes, has the potential to lead to associated biases. Our study further emphasises the lack of publicly available African data for ancestrally relevant validations.

Genomic instability, defined as the acquisition of small to complex genomic variation, is a hallmark of tumourigenesis and in turn telomere shortening. Here we sought to link TTL to common variables of PCa genomic instability, from recurrently mutated cancer driver genes to our all-variant type genome-wide taxonomy. Shared between the ancestries, we found shortened TTL to be associated with acquired variation within the DNA replication tumour-associated gene *SETBP1*. Having previously reported *SETBP1* to be significantly mutated in African-derived tumours^9^, this new PCa driver^36^ showing favourable outcomes in response to immune checkpoint inhibitor treatment in melanoma patients^37^, warrants further investigation on the clinical impact for patients presenting with shortened TTL and *SETBP1* mutant prostate tumours. European specific correlations with shortened TTL most notably included: the DNA mismatch repair gene *MSH2*, known to be associated with telomere shortening^38^; *PTEN* and *TP53* tumour suppressor gene deficiencies, consistent with 2012 findings showing their critical roles in telomere dysfunction which aggravates aggressive PCa progression^39^; while the association with the newly described African-predominant PCa driver *DDX11L1*^9^ remains unexplained. Conversely, *FOXA1* which has key roles in mediating PCa carcinogenesis and oncogenesis driven by androgen receptors^40^, was the only African specific driver gene to be significantly associated with TL (defined as TL ratio). Besides *SETBP1*, TL driver gene associations appear to be ancestry specific with potential clinical relevance.

In addition to single oncogenic drivers, TTLs and TL ratios show significant correlations with genomic instabilities (PGA, Gain and GMS) in both Europeans positively and Africans negatively with no significant ethnic disparities observed in FDRs, where TTLs and TL ratios are robust determinants of higher levels of most types of genomic instability (PGA, TMB, somatic SNV, somatic indel, Gain, Loss, GMS, DBS and SV) based on *P*-values observed in African patients. Shorter TTLs and TL ratios are associated with elevated genomic instabilities in African men, which emphasizes the brutal roles of genomic variations in aggressive PCa in men of African ancestry^9^. Our team has generated an ancestry-defined taxonomy of PCa (GMS classification) based on all types of genomic variation, however excluding for TL data^9^. This study shows our TTL and TL ratio significantly associated with GMS subtypes and raises the TL application as additional genomic parameters for precise PCa classification in the context of ethnic differences.

## Conclusions

We used clinically, technically and analytically matched WGS data from 117 treatment naïve men of African *versus* 62 of European ancestries to investigate the role of TLs in driving PCa within the context of genomic instabilities and clinical presentations, providing further validation using public non-African data. Our study has found that the shortened TTL is an indicator of more aggressive PCa with elevated genomic instabilities in men of African ancestry. European men with shorter BTLs and TTLs were shown to have higher relapse risks with rapid tumour development, where TTLs and TL ratios were found to have associations with more PCa driver genes than African men. These findings provide insight into the essential roles of TLs in driving PCa genomic and clinical disparities and inform future PCa genomic studies, which contribute to the development of diagnosis, precision oncology, prognosis for PCa patients with risk stratifications, especially in ethnically diverse communities.

### Supplementary Information

Supplementary Information.

## Data Availability

Data used in this study was published by Jaratlerdsiri et al.^9^, and made accessible via the European Genome-Phenome Archive (EGA; https://ega-archive.org) under study accession EGAS00001006425 and dataset accession EGAD00001009067 (Southern African Prostate Cancer Study, SAPCS) and EGAD00001009066 (Garvan/St Vincent’s Prostate Cancer Study).
