# Supplementary material for: The impact of telomere length on prostate cancer aggressiveness, genomic instability and health disparities
Source: Sci Rep. 2024 Apr 2;14:7706. doi: 10.1038/s41598-024-57566-1 (PMC10987561; doi:10.1038/s41598-024-57566-1)
Supplement: Supplementary file 1 — Supplementary Information. [file 41598_2024_57566_MOESM1_ESM.docx]

**The impact of telomere length on prostate cancer aggressiveness, genomic instability and health disparities**

Ruotian Huang, M.S. Riana Bornman, Phillip D. Stricker, Ilma Simoni Brum, Shingai B.A. Mutambirwa, Weerachai Jaratlerdsiri and Vanessa M. Hayes

**SUPPLEMENTARY INFORMATION**

**Supplementary Tables**

**Table S1**. Geo-ancestral and clinicopathological features of study cohort.

| Features | | Prostate cancer patients | |
| --- | --- | --- | --- |
| Ancestral origin | | African | European |
| Cohort number | | 117 | 62 |
| Geographical origin | Australia | 0 | 53 |
|  | Brazil | 1 | 4 |
|  | South Africa | 116 | 5 |
| Mean age ± standard deviation  (range) | | 67.060 ± 8.329  (45 – 99)^1^ | 62.241 ± 6.321  (46 – 78) |
| PSA (ng/mL)^2^ | 0 – 9.99 | 11 | 40 |
|  | 10 – 19.99 | 12 | 17 |
|  | 20 – 500 | 73 | 5 |
|  | > 500 | 14 | 0 |
| ISUP grading^3^ | High (3 – 5) | 83 | 54 |
|  | Low (1 – 2) | 30 | 7 |
|  | No (0) | 2 | 1 |

^1^One South African patient of African ancestry was not recorded age.

^2^Seven South African patients of African ancestry were not recorded PSA level.

^3^Two South African patients of African ancestry were not recorded ISUP grading.

**Table S2.** Details of optimal TL cut-offs in European BCR and metastasis cohorts.

| Clinical Outcomes | | Short BTL | Long BTL | Short TTL | Long TTL |
| --- | --- | --- | --- | --- | --- |
| BCR | Mean ± standard deviation | 2828.298 ±  218.285 bp | 3758.199 ±  443.127 bp | 2421.961 ±  261.572 bp | 3566.665 ±  649.352 bp |
|  | Range | 2384.698 –  3192.148 bp | 3276.430 –  4979.750 bp | 1816.568 –  2848.052 bp | 2874.085 –  5153.815 bp |
|  | Cohort Number | 36 | 17 | 18 | 35 |
| Metastasis | Mean ± standard deviation | 2841.508 ±  207.177 bp | 3756.625 ±  338.587 bp | 2640.870 ±  384.443 bp | 4021.605 ±  627.265 bp |
|  | Range | 2447.072 –  3192.148 bp | 3288.910 –  4403.118 bp | 1816.568 –  3311.560 bp | 3327.870 –  5153.815 bp |
|  | Cohort Number | 28 | 11 | 25 | 14 |
| BCR (validation) | Mean ± standard deviation | 3370.282 ±  389.559 bp | 5033.766 ±  1348.707 bp | 1740.030 ±  272.442 bp | 3490.110 ±  781.914 bp |
|  | Range | 2097.895 –  3870.685bp | 3914.157 –  14989.210 bp | 1032.885 –  1982.288 bp | 2021.933 –  6452.088 bp |
|  | Cohort Number | 67 | 223 | 11 | 279 |

Note – BCR and Metastasis included the European cohort from this study, BCR (validation) using the European validation cohort.

**Table S3**. Associations between sequencing impacts and TL.

| Sequencing impacts | BTL | TTL |
| --- | --- | --- |
| Sequencing coverage (SC) | 0.188 | 0.687 |
| Tumour purity (TP) | - | 0.714 |
| Ploidy (P) | - | 0.144 |
| SC + TP + P | - | 0.883 + 0.746 + 0.146 |

Note – *P*-values calculated by one-way ANOVA of BTL and TTL with three sequencing and tumour quality assessments, sequencing coverage, tumour purity, and ploidy with age adjustment.

**Table S4**: Associations between 13 genomic impacts and 35 driver genes, with age adjustment, and tumour TL and ratio using *P*-values by ancestry.

| Genomic impacts | African (n = 117) | | European (n = 62) | |
| --- | --- | --- | --- | --- |
|  | **TTL** | **TL ratio** | **TTL** | **TL ratio** |
| PGA | **1.716e-03** | **1.660e-03** | **1.184e-04** | **8.820e-05** |
| TMB | **0.031** | **4.885e-02** | 0.568 | 0.156 |
| Somatic SNV | **0.028** | **4.945e-02** | 0.560 | 0.220 |
| Somatic indel | **0.037** | **4.988e-02** | 0.473 | **0.020** |
| Somatic SV | 0.370 | 0.242 | 0.307 | 0.665 |
| Gain | **1.369e-03** | **2.645e-03** | **7.167e-03** | **1.769e-03** |
| Loss | **8.517e-03** | **4.464e-03** | **3.959e-05** | **1.041e-04** |
| GMS | **1.090e-07** | **3.480e-10** | **6.520e-08** | **4.310e-10** |
| Clonality | 0.495 | 0.338 | 0.219 | 0.136 |
| SBS | 0.208 | 0.387 | 0.870 | 0.812 |
| DBS | **0.011** | **0.048** | 0.287 | 0.644 |
| ID | **0.020** | 0.153 | 0.950 | 0.358 |
| SV | **3.275e-03** | **7.644e-03** | 0.418 | 0.472 |
| *TMPRSS2* | 0.182 | 0.297 | 0.419 | 0.561 |
| *ERF* | 0.205 | 0.224 | *NA* | *NA* |
| *RB1* | 0.681 | 0.675 | **0.0402** | 0.077 |
| *PTEN* | **0.043** | 0.101 | **2.589e-03** | **0.029** |
| *FAT1* | 0.440 | 0.379 | 0.470 | 0.298 |
| *THADA* | 0.137 | 0.171 | 0.274 | 0.444 |
| *LSAMP* | 0.914 | 0.452 | 0.976 | 0.635 |
| *FOXA1* | **0.041** | **2.706e-03** | 0.259 | 0.135 |
| *TP53* | **0.016** | **0.030** | **3.186e-03** | **0.021** |
| *KMT2C* | **0.011** | 0.055 | 0.353 | 0.833 |
| *SPOP* | 0.080 | 0.063 | **0.019** | **0.020** |
| *MSH2* | 0.959 | 0.862 | **3.276e-04** | **2.673e-04** |
| *BRAF* | 0.111 | 0.157 | 0.159 | 0.507 |
| *MYC* | 0.548 | 0.884 | 0.378 | 0.518 |
| *ZMYM3* | 0.110 | 0.314 | 0.211 | 0.556 |
| *SETBP1* | **9.236e-04** | **4.462e-03** | **2.570e-08** | **1.050e-09** |
| *ZFHX3* | 0.279 | 0.731 | 0.822 | 0.846 |
| *CDK12* | 0.265 | 0.247 | 0.344 | 0.143 |
| *DDX11L1* | **0.019** | **0.014** | **1.700e-06** | **3.350e-05** |
| *STK19* | 0.760 | 0.259 | 0.131 | **8.069e-03** |
| *NCOA2* | 0.336 | 0.258 | 0.365 | 0.170 |
| *PCAT1* | 0.560 | 0.265 | 0.662 | 0.708 |
| *MSH6* | *NA* | *NA* | 0.994 | 0.912 |
| *PAPSS2* | *NA* | *NA* | 0.120 | 0.879 |
| *MTCH2* | 0.866 | 0.619 | 0.950 | 0.664 |
| *ATR* | 0.557 | 0.841 | *NA* | *NA* |
| *BRCA1* | 0.068 | 0.081 | *NA* | *NA* |
| *POLE* | 0.243 | 0.379 | *NA* | *NA* |
| *TP53BP1* | 0.777 | 0.884 | *NA* | *NA* |
| *ATM* | **0.012** | 5.044e-02 | *NA* | *NA* |
| *BRCA2* | **0.029** | 0.055 | *NA* | *NA* |
| *ERCC5* | 0.313 | 0.482 | *NA* | *NA* |
| *ERF* | 0.205 | 0.224 | *NA* | *NA* |
| *PIK3CA* | **0.027** | 0.067 | *NA* | *NA* |
| *APC* | 0.240 | **0.040** | *NA* | *NA* |

Note – *P*-values calculated by one-way ANOVA. *NA*: data not available.

**Table S5**: Associations between four genomic impacts, with age adjustment, and tumour TL and ratio using *P*-values from the 321 European validation cohort.

| Genomic impacts | European Validation (n = 321) | |
| --- | --- | --- |
|  | **TTL** | **TL ratio** |
| PGA | **5.393e-04** | **0.041** |
| Somatic SNV | **1.077e-05** | **0.018** |
| Somatic Indels | **6.232e-05** | **4.315e-03** |
| Somatic GRs | 0.669 | **1.843e-05** |

Note – *P*-values calculated by one-way ANOVA.

**Supplementary Figures**


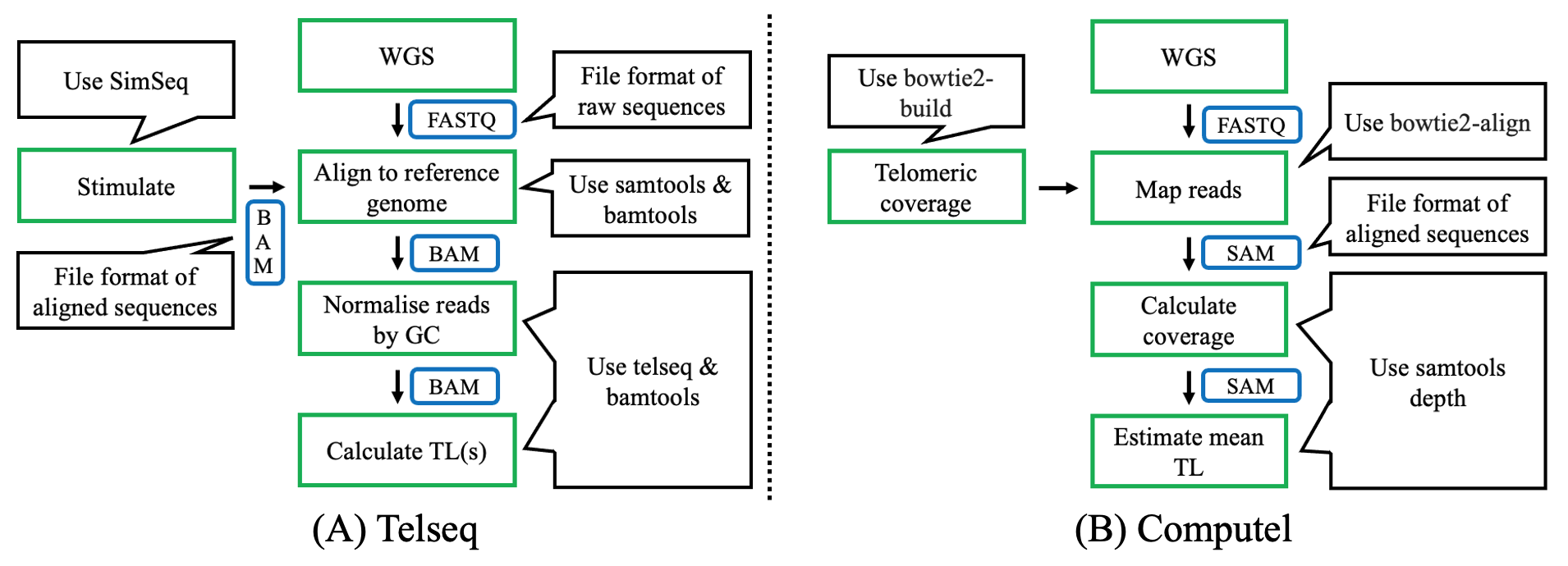


**Figure S1**: Schematic workflow of Telseq (**A**) and Computel (**B**).


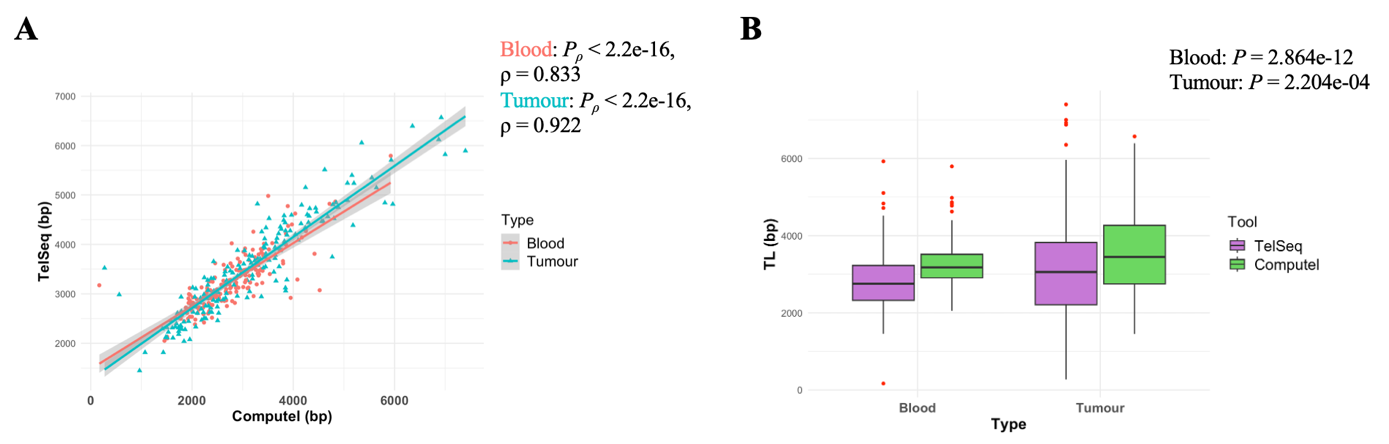


**Figure S2**: *P*-values are from Spearman’s correlation of 179 BTL and TTL estimates by TelSeq and Computel (**A**). Performance comparisons between TelSeq and Computel for the BTL and TTL (**B**), *P*-values from Mann-Whitney U Test. Red dots are outliers.


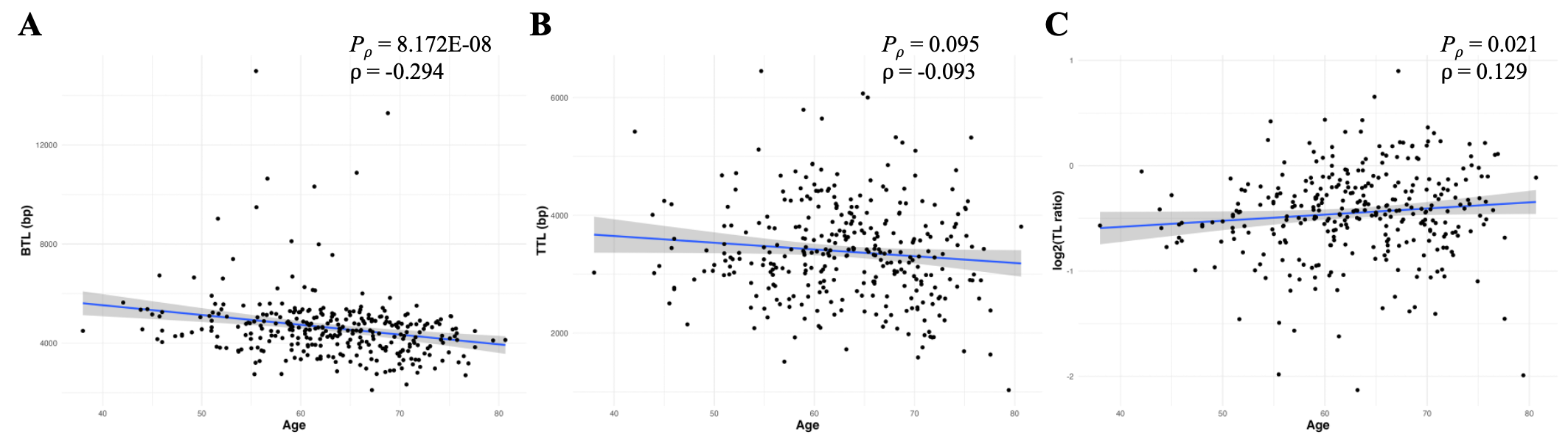


**Figure S3**: Correlations of BTL (**A**), TTL (**B**), and TL ratio (**C**) with age for 321 patients from the European validation cohort, with *P*-values derived from Spearman’s correlation.


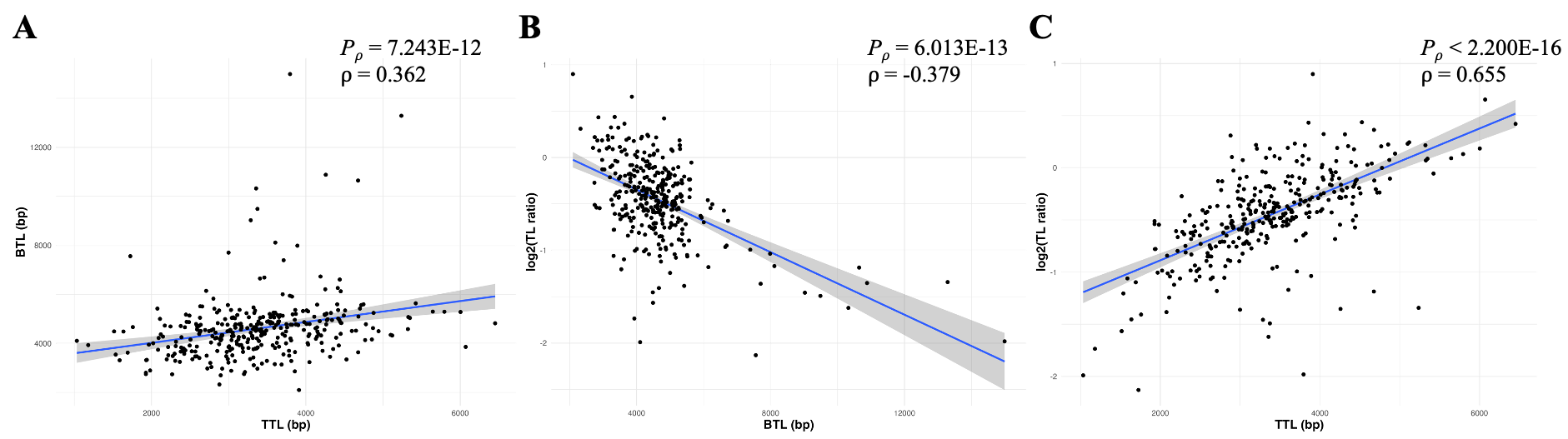


**Figure S4**: Spearman’s correlations of BTL and TTL estimates (**A**), TL ratios in log_2_ transformation and BTL (**B**), and TL ratios in log_2_ transformation and TTL (**C**) for 341 patients from the European validation cohort.


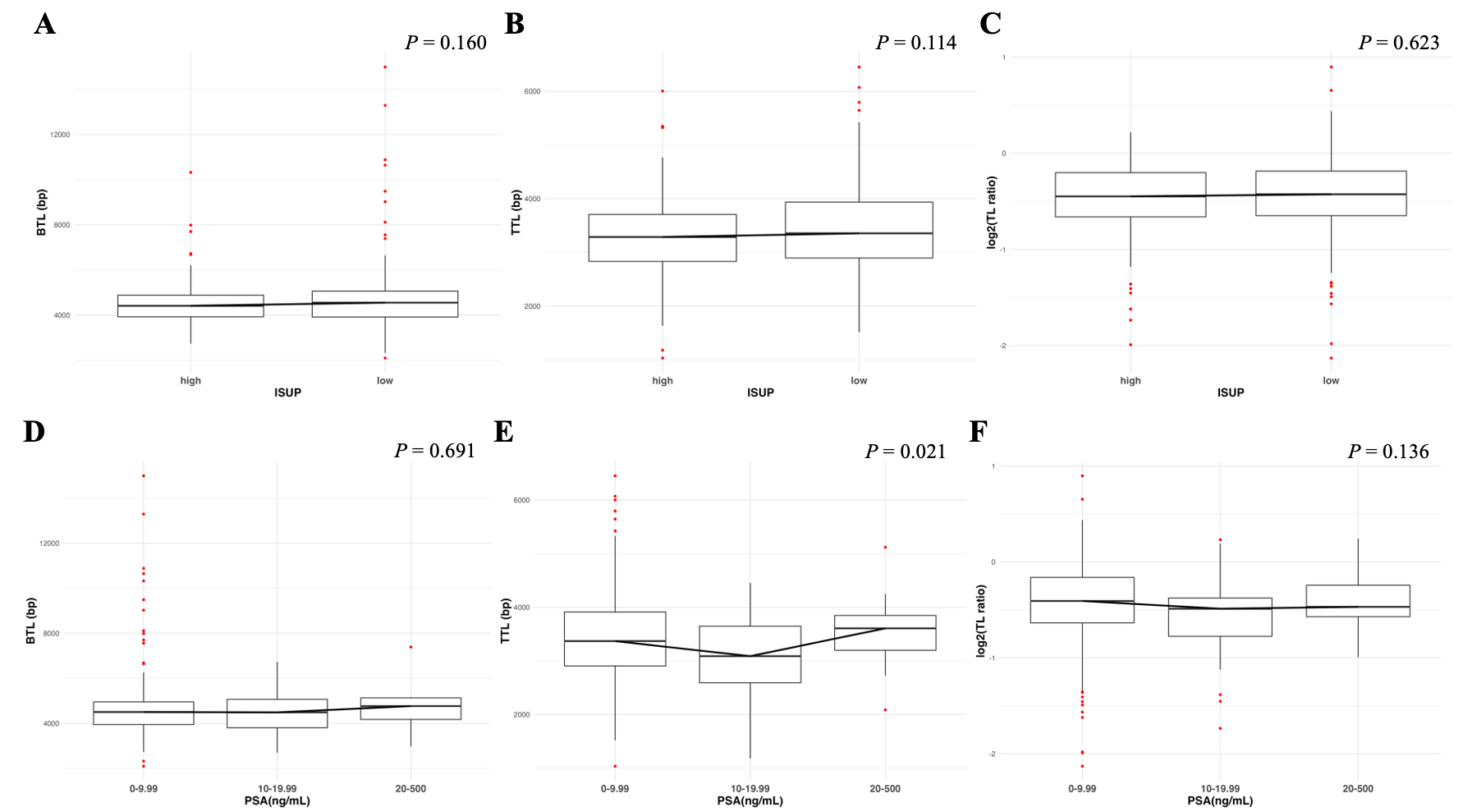


**Figure S5**: Correlations of BTL (**A**), TTL (**B**) and TL ratios (**C**) between low (1-2) and high (3-5) ISUP Grading Groups in 341 patients from the European validation cohort. *P-*values are from one-way ANOVA with age adjustment. Correlations of BTL (**D**), TTL (**E**) and TL ratios (**F**) with the following PSA levels (ng/mL): 0-9.99, 10-19.99 and 20-500 in 336 patients. *P*-values from one-way ANOVA with age adjustment.
